# Preparation of high precision multilayer scaffolds based on Melt Electro-Writing to repair cartilage injury

**DOI:** 10.7150/thno.47909

**Published:** 2020-08-13

**Authors:** Yu Han, Meifei Lian, Binbin Sun, Bo Jia, Qiang Wu, Zhiguang Qiao, Kerong Dai

**Affiliations:** 1 Department of Orthopaedic Surgery, Shanghai Key Laboratory of Orthopaedic Implants, Shanghai Ninth People's Hospital, Shanghai Jiao Tong University School of Medicine, Shanghai 200011, China.; 2 Clinical and Translational Research Center for 3D Printing Technology, Shanghai Ninth People's Hospital, Shanghai Jiao Tong University School of Medicine, Shanghai, 200011, China.; 3 Department of Prosthodontics, Shanghai Ninth People's Hospital, College of Stomatology, Shanghai Jiao Tong University School of Medicine, National Clinical Research Center for Oral Diseases, Shanghai Key Laboratory of Stomatology & Shanghai Research Institute of Stomatology, Shanghai 200011, China.

**Keywords:** Melt Electro-Writing, Inkjet, 3D print, cartilage, chondrogenic differentiation

## Abstract

**Rationale:** Articular cartilage injury is quite common. However, post-injury cartilage repair is challenging and often requires medical intervention, which can be aided by 3D printed tissue engineering scaffolds. Specifically, the high accuracy of Melt Electro-Writing (MEW) technology facilitates the printing of scaffolds that imitate the structure and composition of natural cartilage to promote repair.

**Methods:** MEW and Inkjet printing technology was employed to manufacture a composite scaffold that was then implanted into a cartilage injury site through microfracture surgery. While printing polycaprolactone (PCL) or PCL/hydroxyapatite (HA) scaffolds, cytokine-containing microspheres were sprayed alternately to form multiple layers containing transforming growth factor-β1 and bone morphogenetic protein-7 (surface layer), insulin-like growth factor-1 (middle layer), and HA (deep layer).

**Results:** The composite biological scaffold was conducive to adhesion, proliferation, and differentiation of mesenchymal stem cells recruited from the bone marrow and blood. Meanwhile, the environmental differences between the scaffold's layers contributed to the regional heterogeneity of chondrocytes and secreted proteins to promote functional cartilage regeneration. The biological effect of the composite scaffold was validated both *in vitro* and *in vivo*.

**Conclusion:** A cartilage repair scaffold was established with high precision as well as promising mechanical and biological properties. This scaffold can promote the repair of cartilage injury by using, and inducing the differentiation and expression of, autologous bone marrow mesenchymal stem cells.

## Introduction

Osteoarthritis (OA) is currently a major research priority in Orthopedic Medicine due to its global prevalence and associated economic impact. In China, specifically, billions of dollars each year are allocated to medical funding for OA. The poor self-repair capacity of articular cartilage due to its lack of a blood supply and stem cells, contributes to the need for medical interventions to treat OA. Clinically, arthroplasty is used to treat patients with severe cases of OA. In contrast, mild to moderate cases, including partial cartilage exfoliation, are treated via arthroscopy, which can also be supplemented with microfracture or mosaic grafting to partially reconstruct the cartilage. However, the cartilage formed by simple microfracture is primarily composed of collagen type I and does not, therefore, exhibit good wear resistance 1-3. Meanwhile, mosaic grafting also tends to incur damage in the donor tissue. Therefore, recent research has focused on developing strategies to induce repair and reconstruction of healthy cartilage. Specifically, tissue engineering technologies that provide a repair scaffold combined with stem cells and biological differentiation factors provide a feasible method for promoting cartilage regeneration.

However, the non-uniform structure of articular cartilage poses a unique challenge for designing regeneration approaches. In fact, cartilage is composed of multiple layers, each with its own unique composition and associated function. For example, the outer layer of the cartilage provides lubrication to reduce joint friction. The middle layer exhibits elastic properties to allow it to play a supportive role, while the deep layer is connected to the subchondral bone facilitating anchoring of the tissue. Due to the limitations of traditional technology, preparation of tissue engineering scaffolds with unique structures and distribution patterns has proven challenging. However, the emergence of 3D printing technology has served to improve this issue 4-6.

3D printing technology can generate different scaffolds by switching between printing inks to accurately fill specific structures of the scaffold with appropriate materials, allowing for the preparation of biological scaffolds with complex structures suitable for repairing different tissues in an integrated manner 7. Hence, 3D printing technology is increasingly being applied throughout the medical field. For instance, recently, many studies have described the generation of layered cartilage scaffolds, including those using hydrogels 8-11. However, since the cartilage tissue is relatively thin, the thickest portion being 3 mm at the femoral condyles, current printing technology is not capable of printing multi-layered thin scaffold to accurately mimic the complexity of healthy cartilage tissue 12-14.

The Melt Electro-Writing (MEW) technology may address this limitation of current printing methods in that it functions to increase the electric field based on ordinary fused deposition molding (FDM), which uses the electric field to pull the extruded material, and reduces the fiber diameter to improve printing accuracy. In this way, MEW has demonstrated higher printing accuracy than fused deposition manufacturing (FDM) or melt extrusion printing 15. In fact, the printed fiber can be as thin as several micrometers in diameter within the controllable range, which reflects the structural dimension of healthy tissues and enables simulation of complex cartilage tissues 16-19.

However, although MEW can improve the accuracy of printed objects, it is not yet capable of mimicking the zonal heterogeneity of cartilage tissue by using different materials in different areas 20, 21. Moreover, the materials currently available for MEW are relatively limited, as not all biological materials can be used for printing. To address this issue, Inkjet technology was employed in the current study to spray different pre-packaged cytokine-containing microspheres onto the scaffold. Specific cytokines are important participants in the formation and repair of cartilage tissue. Hence, their addition to the damaged area can promote cartilage repair and the formation of tissue-specific proteins. Hence, Inkjet and MEW were printed alternately to construct a multi-layered composite scaffold capable of firmly adhering to the tissue and mimicking the complexity of normal cartilage, while also improving regeneration accuracy.

Transforming growth factor-beta 1 (TGF-β1) is a key regulator of cartilage differentiation in bone marrow mesenchymal stem cells (BMSCs). Hence, its use as a cell culture supplement to promote the formation of cartilage from BMSCs, is recognized as the key to maintaining cartilage differentiation *in vitro* 22, 23. Meanwhile insulin-like growth factor-1 (IGF-1) can promote the role of TGF-β1 and enhance production of type II collagen and aggrecan 24. Moreover, bone morphogenetic protein-7 (BMP-7) promotes secretion of PRG4 by chondrocytes, which participates in lubrication of the cartilage surface 25, 26; and hydroxyapatite (HA) promotes the differentiation of chondrocytes into deep cartilage 27. We, therefore, loaded TGF-β1 in the full layer of the stent, and BMP-7, IGF-1, and HA in the surface layer, middle layer, and deep layer, respectively, with the goal of promoting cartilage injury repair.

Moreover, currently there is no agreed upon consensus regarding the necessity of loading cells in the scaffold. Some scholars believe that loading cells can increase the repair effect of the scaffold. Hence, cells can be loaded into the scaffold through a certain period of *in vitro* culture. Alternately, hydrogels can be used to directly coat the cells and cytokines onto the scaffold before implanting into the body to promote cartilage repair 28-30. However, this method requires the preparation of autologous cells, which can be time consuming. Moreover, while using allogeneic cells may reduce the required time for scaffold generation, there is a high associated risk of rejection and the level of accuracy associated with this method is not optimal. Therefore, here we describe the application of a cell-free scaffold containing cytokines packaged into microspheres loaded on the scaffold surface for slow-release. Combined with the microfracture commonly used in clinical practice, following implantation, the scaffold relies on the body's stem cells to facilitate tissue regeneration. Meanwhile the slow release of cytokines promotes cell adhesion, proliferation, and differentiation into chondrocytes, and other cell types, in different regions of the scaffold. Thus, the resulting tissue composition resembles that of normal cartilage, achieving successful cartilage damage repair.

## Methods

### Preparation of microspheres

The poly D,L-lactic-co-glycolide (PLGA) microspheres were prepared using the double emulsifycation method, as follows. First, 0.1 g of polyvinyl alcohol (PVA) was dissolved in 100 mL of water and 0.1 g of PLGA (Sigma-Aldrich, USA) was dissolved in 1 mL methylene dichloride. The Tween and PLGA dichloromethane solutions were then cooled in an ice bath. A total of 500 μL of PBS was added to the PLGA dichloromethane solution and dispersed using an ultrasonic cell disrupter for 10 s until the liquid became milky white. This solution was then added to the Tween solution dropwise, ultrasonically dispersed for an additional 2 min until the liquid was pale milky white, and then placed on a magnetic stirrer for 4 h to volatilize the methylene chloride organic solvent. After the microspheres were formed, the solution was centrifuged at 2500 *× g* for 5 min; the supernatant was discarded. The pellet was then washed thrice with deionized water via centrifugation. The pellet was frozen at -20 °C, and empty PLGA microspheres were obtained via vacuum drying.

### Loading of PLGA microspheres with BSA, TGF-β1, BMP-7, and IGF-1

To optimize the loading of TGF-β1, BMP-7, and IGF-1 (Cyagen, China) in PLGA microspheres, we chose BSA as the mimic protein. The BSA/PLGA microspheres were prepared as follows. A solution of 100 µg BSA in 50 µL PBS was mixed with 1 mL 10% PLGA solution. BSA and cytokine-containing PLGA microspheres were then prepared following the method described above in preparation of microspheres. To avoid cytokine inactivation, the prepared PLGA microspheres were stored at -20 °C until use.

### Determination of productive rate and coating rate of BSA/PLGA microspheres

All BSA containing PLGA microspheres made using 100 mg PLGA (n = 4) were lyophilized and weighed. A total of 10 mg of prepared BSA/PLGA microspheres (n = 4) was dissolved in 0.9 mL of a mixed solution of 1 mol/L NaOH and 0.1 mL of PBS stirred at 21 °C for 2 h; this solution was then mixed with 1 mL 0.9 mol/L HCl solution. A 20 μL sample of this solution was used to determine BSA content using a BCA protein test kit (Biyuntian, Shanghai), following the manufacturer's instructions. The BSA coating rate (%) was calculated as follows: quantity of BSA (μg)/theoretical quantity of BSA (10 μg) × 100 (%).

### Preparation of the multilayer scaffolds

A total of 20 g of polycaprolactone (PCL) (Mn 45000, Sigma-Aldrich, USA) was placed in the printing barrel and melted at 65 °C. A 21G printing needle was connected to the barrel, which was then placed in the electrostatic direct-write printer. The needle was connected to negative electricity, and printing pressure adjusted to 0.1 MPa. The distance between the needle and the table was 2-4 mm. The voltage setting for the initial 2 mm of printing was 3 kV, which was increased by 1.5 kV for every additional 1 mm. After printing ten layers, the barrel was replaced with the microsphere-containing barrel.

The cytokine-loaded microspheres (10 mg) were evenly dispersed in 10 mL of pure water and placed in the printing barrel, and a 30 G needle was connected. The pressure and voltage were changed to 0.01 MPa and -2.5 kV, respectively. The microspheres were then sprayed using the Inkjet method, after which it was switched back to a PCL barrel and printed in alternate layers. From top to bottom, the top 20 layers of the outer surface were sprayed with TGF-β1/PLGA and BMP-7/PLGA microspheres; the next 30 layers in the middle were sprayed with TGF-β1/PLGA and IGF-1/PLGA microspheres; finally, PCL mixed with HA was printed for the bottom 100 layers, and TGF-β1/PLGA microspheres were sprayed.

After printing, a mixture of n-hexane and tetrahydrofuran (9:1 v/v) was added to the scaffolds to facilitate physical bonding of the PLGA microspheres with the PCL scaffold. The scaffolds were then dried in a drying box under vacuum for a minimum of 24 h to remove the organic solvent.

### Characterization of the scaffold with microsphere

The scaffold samples were fixed on aluminum foil, sprayed with 6 nm thick platinum (OPC-80T, SPI Supplies, West Chester, PA), and observed under a scanning electron microscope (SEM) (LEICA, Germany). To clarify the effect of the solvent treatment, the scaffolds were soaked in PBS with continuous shaking at 100 rpm for 5 min, and the microspheres were examined to determine if they had remained attached to the scaffolds. Some microsphere samples were prepared by loading FITC dyes. The microsphere-containing scaffolds were then observed and recorded by a fluorescence microscope (LEICA, Germany).

### *In vitro* BSA/PLGA microsphere-PCL composite scaffold degradation and protein release

The composites were weighed and immersed in α-DMEM medium (Gibco, USA) with continuous shaking at 100 rpm and 37 °C. The medium was replaced every week. At each time point, the samples were collected, washed with distilled water, freeze-dried, and weighed. The % weight loss of samples was calculated according to the equation %weight loss = 100 (w_0_ - w_t_)/w_0_, where w_t_ is the dry weight remaining at a given degradation time t, and w_0_ is the initial weight.

A BSA/PLGA Microsphere-PCL composite scaffold containing 10 mg BSA/PLGA microspheres (n = 4) was added to 2 mL PBS (pH = 7.4) with continuous shaking at 100 rpm and 37 °C for the indicated time. At each time point, 1 mL of the solution was removed and replaced by 1 mL of fresh PBS. The concentration of BSA in the samples collected at various time points was determined using a BCA protein assay kit (Biyuntian, Shanghai), according to manufacturer's instructions.

### Scanning electron microscopy

Simple scaffolds, microsphere attached scaffolds, and microsphere adhered scaffolds were examined using SEM (Philips XL-30, Netherlands). Before SEM imaging, samples were cut with a razor blade in liquid nitrogen and sputter-coated with platinum according to a previously published method 31.

### Biomechanical examinations

The biomechanical properties of the samples were tested by a biomechanical analyzer (Instron-5542, USA, n = 5 in each group) according to the manufacturer's guidelines. All samples were exposed to a constant strain rate of 0.5 mm/min until 80% of the maximum deformation was achieved. The elastic modulus and compression modulus were calculated according to the slope of the stress-strain curve 32.

### *In vitro* cell experiments

#### Cell isolation and culture

All experiments were approved by the Animal Ethics Committee of the Ninth People's Hospital affiliated with Shanghai Jiao Tong University. BMSCs were obtained from 2-4-week-old New Zealand white rabbits. The femur and tibia of the rabbits were removed and repeatedly washed with α-DMEM medium (Gibco, USA) containing high glucose α, 10% fetal bovine serum, 1% penicillin-streptomycin, and 100 U/mL heparin sodium, to elute the stem cells from the bone marrow. After centrifuging at 100 ×* g* for 5 min, the pellet was resuspended in α-DMEM medium containing 10% fetal bovine serum, 1% penicillin-streptomycin, 1% insulin-transferrin-selenium, 100 µg/mL sodium pyruvate, 40 µg/mL proline, 10 µg/mL dexamethasone, and 50 µg/mL ascorbic acid. The cells were seeded in a petri dish, and cultured in a 5% CO_2_ incubator at 37 °C. The medium was changed after 2-3 days. The primary BMSCs were recorded as P0.

#### Cell seeding and culture on scaffolds

The scaffold was soaked in 75% ethanol solution for 2 h, followed by three washes in sterile PBS for 5 min each. The scaffolds were divided into four groups: the surface layer (SL) group had scaffolds with 100 µm pores, while the middle layer (ML) and deep layer (DL) groups had scaffolds with 200 µm pores. The fourth group was the blank control. The SL group was loaded with TGF-β1&BMP-7/PLGA microspheres, and the ML group was loaded with TGF-β1 & IGF-1/PLGA microspheres. In the DL group, the PCL contained 10% hydroxyapatite (HA) and was loaded with TGF-β1/PLGA microspheres. The scaffolds were placed in a 6-well plate or 96-well plate. The P2 bone BMSCs were collected from culture following digestion with 0.25% trypsin. After washing via centrifugation, the pellet was resuspended in media at a concentration of 3000 cells/mL with 1 mL of BMSC suspension containing the scaffold added to 6-well plates, or 100 µL to 96-well. The plates were then placed in an incubator for 4 h, and supplemented with 1 mL or 100 μL of culture medium, respectively. The time was recorded, and the media was periodically observed and changed.

#### Cell adhesion and proliferation

The cell counting kit-8 test (Dojindo Molecular Technology, Japan) was used to measure adhesion and proliferation of different scaffold cells. BMSCs were seeded in the scaffolds, as described above. At 4 d, 7 d, 14 d and 21 d, the scaffolds were transferred to a new 96-well plate. Next, 200 μL of medium with 10% CCK-8 reagent was added to each well and placed in the incubator for 1 h in the dark. A total of 100 μL of medium (supernatant) from each well was transferred to a clean 96-well plate, and absorbance was measured with a microplate reader (Thermo Fisher Scientific, USA) at 450 nm (630 nm as a reference).

At 7 d and 14 d, the cell-bearing scaffolds were fixed with 4% paraformaldehyde and incubated with TRITC-labeled phalloidin at room temperature for 45 min. After three 5-min washes with PBS, the scaffolds were incubated with DAPI solution for 10 min at room temperature and observed under a fluorescence microscope (LEICA, Germany).

#### RNA isolation and quantitative reverse transcription-polymerase chain reaction (rt-PCR)

RNA was extracted from samples in different groups (n = 5 per group) using the DirectzolTM RNA MiniPrep kit (Zymo Research, USA) and reverse transcribed into cDNA using the qScriptTM cDNA Synthesis kit (Quanta Biosciences, USA) and a MiniOpticon real-time PCR detection system (BioRad, USA), according to the manufacturer's instructions. Polymerase chain reaction (PCR) was conducted using a PerfeCta SYBR Green FastMix kit (Quanta Biosciences, USA) according to the manufacturer's instructions. The expression genes were analyzed using an ABI 7300 real-time PCR System (Thermo Fisher, USA). The expression of proteoglycan 4 (*PRG4*), cartilage intermediate layer protein 1 (*CILP*), collagen type II (*COL2A1*), collagen type I (*COL1A1*), and SOX-9 (*SOX9*) was normalized to the expression level of the housekeeping gene for β-actin (*ACTB*). The primer sequences for *PRG4, CILP, COL2A1, COL1A1, SOX9*, and *ACTB* are listed in Supplementary Table 1.

#### Immunofluorescent staining

After 21 days of culturing, the cell-bearing scaffolds (n = 4 per group) were fixed in 4% paraformaldehyde for 2 h. Triton-X (0.1%) was used to soak the scaffolds for 5 min, which were then washed thrice with PBS for 5 min each. Blocking was performed with BSA at room temperature for 1 h. After blotting with water-absorbing paper, primary antibodies (anti-PRG4, anti-CILP, anti-COLII, anti-COLI, and anti-SOX-9) (1:100, Abcam, UK) were added to each sample and incubated at 4 °C overnight. Following washes with PBS, a corresponding secondary antibody (1: 500, Abcam, UK) was added, incubated at room temperature for 1 h, and washed with PBS. The scaffolds were then labeled with FITC phalloidin (1:200, MaoKang, China) and incubated at room temperature for 2 h, followed by incubation with DAPI staining solution (1:1000, MaoKang, China) for 30 min at room temperature. After the final wash with PBS, samples were observed and imaged under a confocal microscope (LEICA, Germany). Analysis and quantification of protein expression was performed using ImageJ.

### *In vivo* animal experiment

#### Surgical procedure

All animal operations and experiments were approved by the Animal Ethics Committee of the Ninth People's Hospital affiliated to Shanghai Jiao Tong University. *In vivo* experiments were performed using three-layer composite scaffolds, divided into three groups: 1. Blank control, 2. Simple scaffold, and 3. Cytokine-containing microsphere loaded scaffold (Composite scaffold). Six-month-old adult male New Zealand white rabbits were used, weighing approximately 3-4 kg., housed at 27 °C room temperature, with a 12 h light/dark cycle, and 55-65% humidity. Animals were anesthetized using 50 mg/kg ketamine and 50 mg/kg xylazine. A midline incision of the right knee was then performed, and the medial collateral ligament was cut from inside the patella to enter the joint cavity. A 4 mm diameter and 2 mm deep well was drilled into the femoral condyle. A 1 mm diameter Kirschner wire was used to penetrate the subchondral bone. The scaffolds were then implanted into the drilled wells. The joint cavity was closed with conventional suturing. The animals were sacrificed at 3, 6, and 12 w post-surgery.

#### Histology and immunofluorescence staining

Cartilage tissue samples were collected at 3, 6, and 12 w after scaffold implantation, and paraffin-embedded sections were prepared following a conventional decalcification process. Standard hematoxylin and eosin (HE; Thermo Scientific, USA) staining was used to evaluate cell distribution and tissue conditions. Sirius red staining (Thermo Scientific, USA) was used to observe collagen formation. For protein expression, COLII, COLI, PRG4, SOX9 antibodies (Abcam, UK) were used as primary antibodies, with incubation overnight at 4 °C, followed by incubation with the respective secondary fluorochrome-conjugated anti-mouse IgG antibodies (Invitrogen, USA) for 2 h in the dark at room temperature. The stained sections were observed and imaged under a fluorescent microscope (LEICA, Germany).

#### Biomechanical and biochemical evaluations of the scaffolds

The compression modulus of samples (n = 4 animals per group at 3 w and 12 w) was tested following the method described in section 2.1.8. The same samples were then used to test the total collagen and COLII content. The total collagen content was quantified using a hydroxyproline assay, and the COLII content was quantified using sandwich ELISA, according to a published method 33-35.

### Statistical analysis

Data analysis was performed using SPSS 25.0 statistical software (SPSS, USA). Quantitative data are expressed as mean ± standard deviation (SD). Data were analyzed by independent sample *t*-test and one-way analysis of variance (ANOVA). P < 0.05 was considered statistically significant.

## Results

### Preparation of the composite scaffold

#### Preparation of microspheres

**Figure 1:**
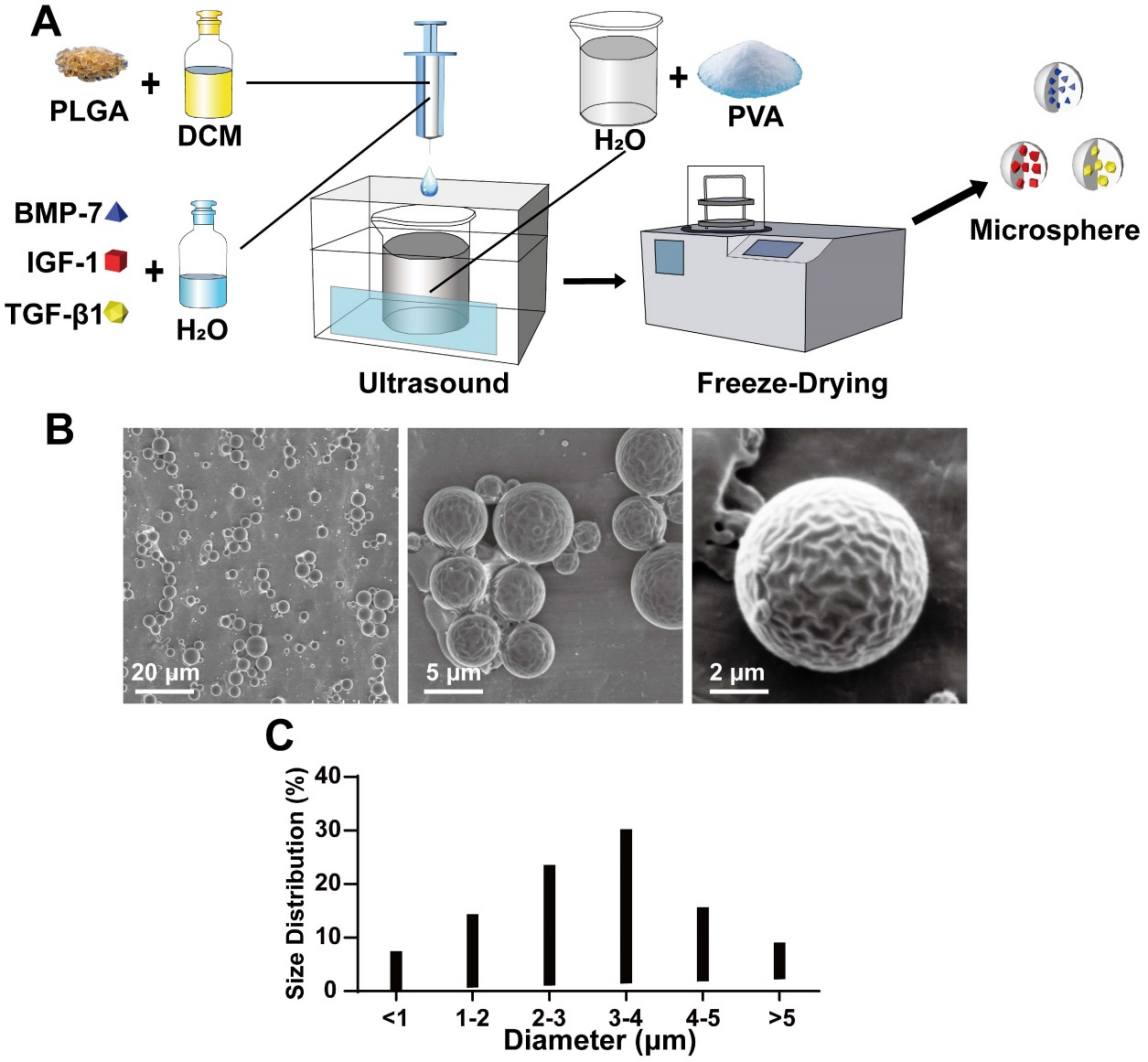
** Production and physical properties of microspheres. A)** Schematic diagram of the double emulsification method for microspheres production; **B)** Scanning electron microscope (SEM) morphology of microspheres; **C)** Statistics of microsphere diameter distribution.

First, to be able to load the cytokines on the scaffold with relative stability for a sustained period, we used PLGA microspheres to encapsulate the cytokines via the double emulsification method, as shown in Figure 1A. The synthesized microspheres were imaged under an electron microscope (Figure 1B) and found to be of relatively uniform shape and size (Figure 1B). The particle size analysis showed that the microsphere particle size was well controlled, with 1-5 μm diameter microspheres accounting for 83.6% of the total, and 3-4 μm size microspheres accounting for 30.2% (Figure 1C). Next, the effect of microsphere loaded protein was verified using BSA as the model protein for loading and analysis. The results showed that the BSA content per 10 mg microspheres was approximately 7.373 ± 1.013 μg.

#### Fabrication of simper scaffold and Inkjet of microsphere

**Figure 2:**
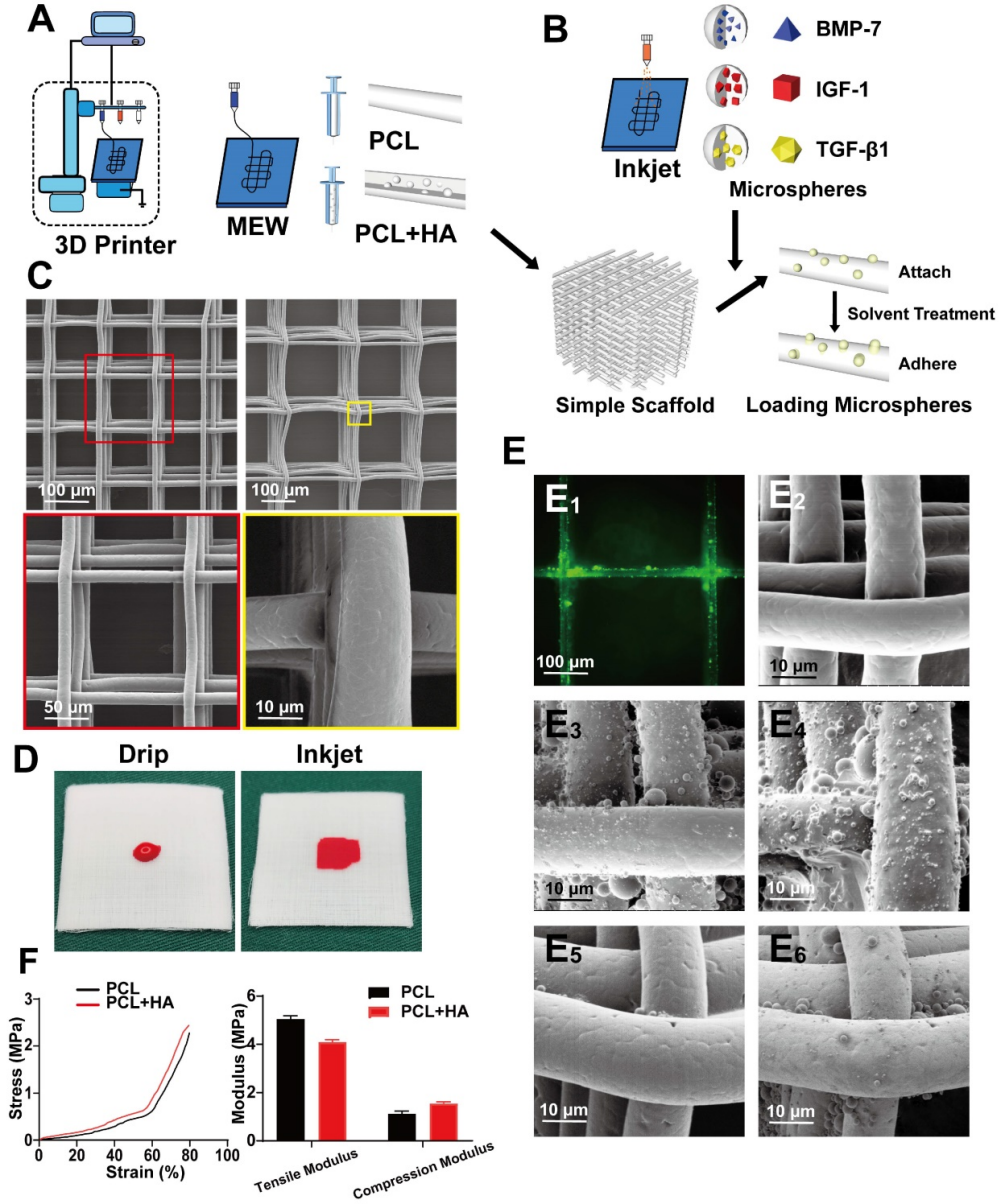
** Fabricating a composite scaffold via alternate printing with MEW and Inkjet. A and B)** MEW and Inkjet alternately print to prepare composite scaffold; **C)** The appearance of the simple PCL scaffold observed under the electron microscope at low magnification and high magnification; **D)** Comparative gross view of liquid and Inkjet; **E)** image of green (FITC) fluorescent microspheres adhering to the scaffold (E1), and comparison of SEM images of a simple scaffold (E2), microsphere attached scaffold (E3) and microsphere adhered scaffold (E4); after being soaked and washed in PBS, microsphere attached scaffold (E5) and microsphere adhered scaffold (E6). **F)** Compression modulus test curve and quantitative analyses of scaffold mechanical properties.

During the preparation process, cytokine-loaded microspheres were sprayed to make the scaffold and microspheres composite. Pure PCL was used for the SL and ML, and 10% HA-PCL was used for DL (Figure 2A). The cytokines included were, BMP-7 for the SL, IGF-1 for the ML, and TGF-β1 for the entire scaffold (Figure 2B). The shape of a common scaffold was observed via electron microscopy. To maintain the overall structure and maintain the internal environment, the SL was prepared with a 100 µm pore structure, while the middle and DLs used a 200 µm structure. Furthermore, the fiber shape was even, and the surface was smooth (Figure 2C).

Next, Inkjet was used to uniformly spray the microspheres at the required positions. This method ensured accurate compounding of the microsphere and PCL layer in appropriate positions. Due to the hydrophobic nature of the PCL, the dropwise addition of the microsphere suspension would not have achieved compounding with PCL (Figure 2D), which allows the microspheres to attach to the scaffold surface. The microspheres then adhered to the surface of the scaffold through the organic solvent in a slightly soluble manner, so facilitate firmer fixation. Furthermore, the scaffold was labeled with FITC to better visualize the adherence of microspheres to the PCL (Figure 2E1). Attachment of the scaffold, and adherence of microspheres was observed on the simple scaffold (Figure 2E2), after spraying the microspheres (Figure 2E3), and after solvent treatment (Figure 2E4). After soaking and washing in PBS, the solvent-treated scaffold (Figure 2E6) exhibited superior microsphere adhesion on the surface compared to the untreated scaffold (Figure 2E5). The mechanical properties of the 100 layers (approximately 1 mm) containing 200 μm pore scaffolds composed of pure PCL or PCL with 10% HA were then tested. The results showed that after adding 10% HA, the tensile modulus of the scaffolds decreased, while the compression modulus was improved (Figure 2F, Figure S1).

#### Fabrication and testing of composite scaffolds

**Figure 3:**
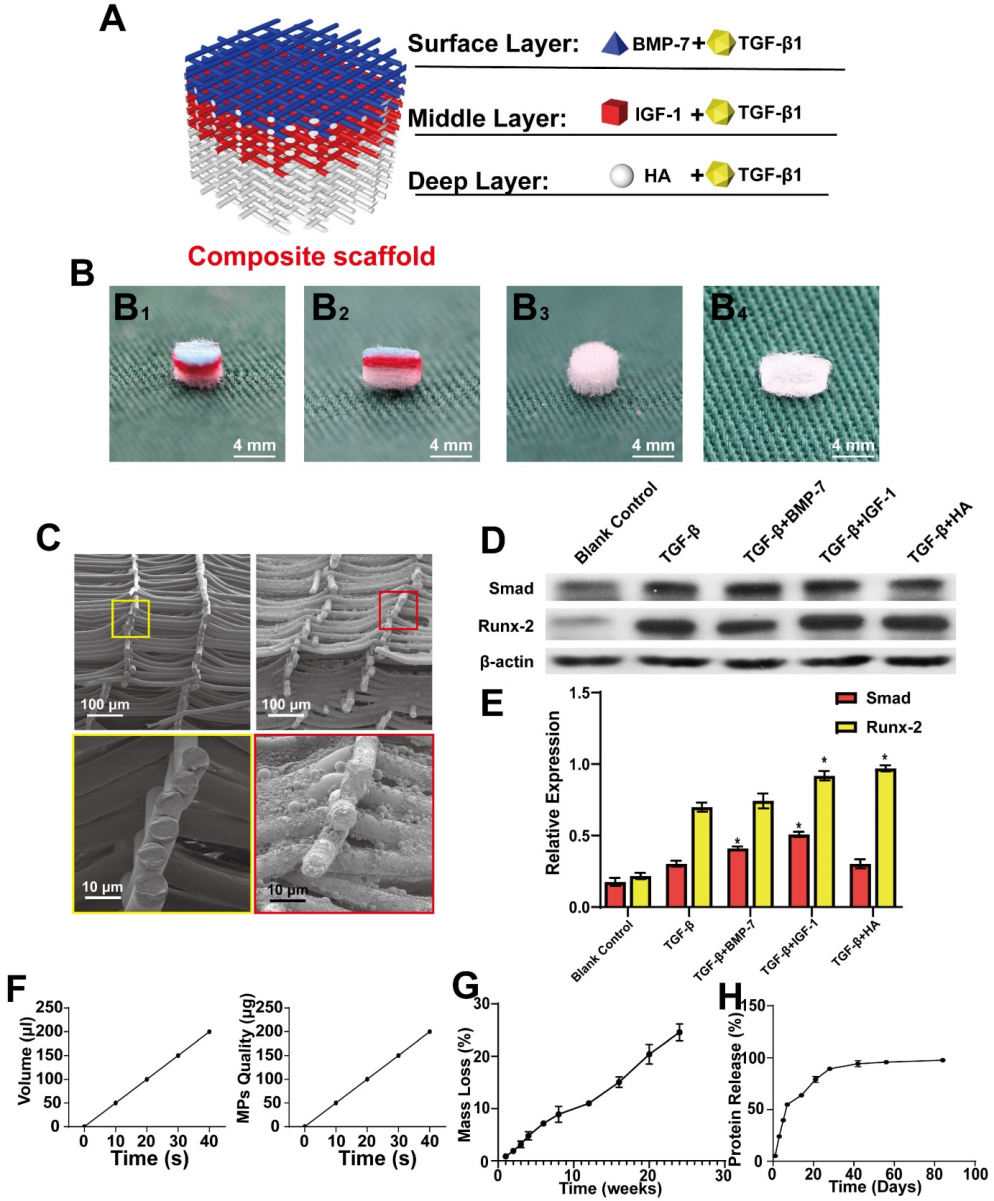
** Design of the composite scaffold. A)** Schematic diagram of the composite scaffold design; **B)** Gross view and cross-section of the scaffold Inkjet by color ink (B1 and B2), Gross view of the front and cross-section of the scaffold (B3 and B4); **C)** Cross-section of a simple scaffold and composite scaffold, as observed through SEM; **D)** Expression of SMAD and RUNX-2 in BMSCs following treatment with different cytokines for 21 days; **E)** Expression levels of SMAD and RUNX-2 quantified using ImageJ software, and the relative intensities were normalized to their respective β-actin expression levels; **F)** Relationship between spray time and spray volume of Inkjet; **G and H)** Degradation and protein release curve of the composite scaffold. (* P < 0.05 compared to blank control and TGF-β group).

MEW and Inkjet printing were performed alternately to build a composite scaffold. The overall view of the composite scaffold is shown in Figure 3A, and the general view is presented in Figure 3B, while the cross-section of a simple scaffold and composite scaffold is shown in Figure 3C. TGF-β was found to promote the expression of SMAD and RUNX2. Meanwhile, BMP-7 and TGF-β combined promoted higher levels of SMAD, which can promote cartilage regeneration. Further, IGF-1 was observed to improve the effect of TGF-β on SMAD. Both HA and IGF-1 were found to increase RUNX expression (Figure 3D-E). Taken together, these results demonstrate that the effects of IGF-1 and HA combined with TGF-β can promote the repair of middle and deep cartilage layers.

In addition, the amount of Inkjet-sprayed microsphere suspension was measured and the relationship between microsphere mass and time was investigated (Figure 3F). For measurement and conversion, 20 s/10 layers was applied, that is, 100 μL of suspension was sprayed with 100 µg microspheres, the speed and path were adjusted, and microspheres were uniformly sprayed on the support. Next, the degradation performance of the composite scaffold was determined, demonstrating that the scaffold could maintain 74.5% of its quality at 24 w. This slow degradation performance of the scaffold would reduce the level of induced changes occurring in the surrounding tissues (Figure 3G).

The BSA microsphere composite scaffold was then used to measure sustained protein release from the scaffold over time, as measured in weeks. (Figure 3H). It was observed that the scaffold released approximately 50% of the protein in approximately 1 week, while the remainder was released slowly.

### *In vitro* experiments

#### BMSC adhesion and proliferation on the scaffold

**Figure 4:**
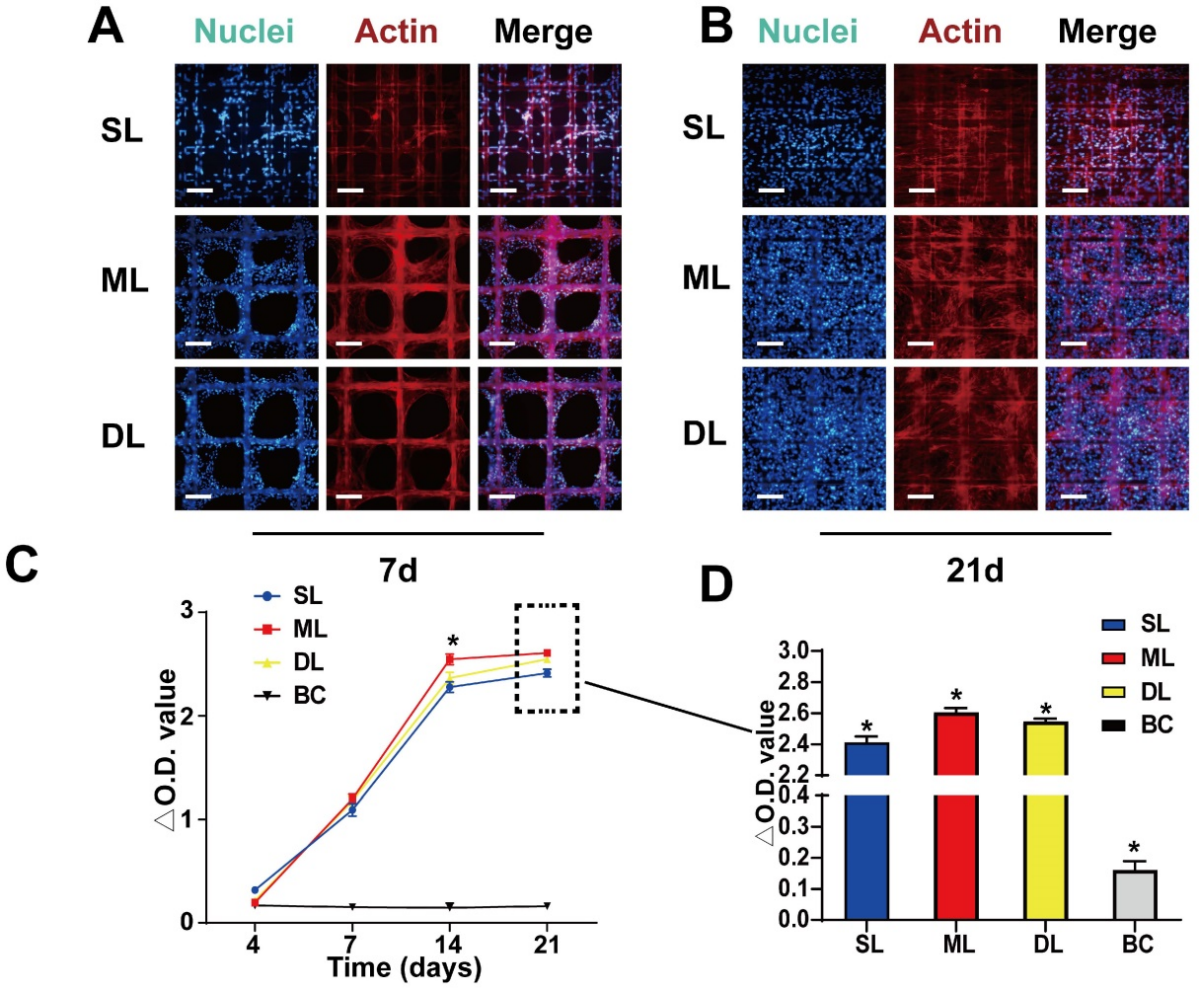
** BMSC adhesion and proliferation on the scaffolds. A, B)** BMSC adhesion and proliferation on scaffolds at 7 d and 21 d. **C, D)** CCK-8 assay results showing proliferation rates of the cells on scaffolds at various time points. (Scale Bar: 100 µm) (*P < 0.05 compared to other groups).

To test the biocompatibility of the scaffold, the adhesion and proliferation of BMSCs was evaluated on the scaffold. Seven days after seeding the cells were observed to be firmly adhered to the scaffold (Figure 4A). Due to the smaller pore size, and larger surface area of the SL scaffold (pore size = 100 μm) compared to the ML and DL scaffolds (pore size = 200 μm), the cells quickly adhered and made contact with each other to form a reticulate. Meanwhile, the cells on the ML and DL scaffolds adhered to the material and exhibited partial proliferation (Figure 4A). After 21 days of seeding, the cells had proliferated and filled the scaffold, showing good biocompatibility of the material and cell viability on scaffolds (Figure 4B). The cell viability, as measured by CCK-8 assay at 4 d, 7 d, 14 d and 21 d after cell seeding, showed that under the influence of growth factors and scaffolds, the ML scaffold cells exhibited the highest activity/viability, although the difference was not particularly obvious between the experimental scaffolds (Figure 4C-D). These results agreed with those of the fluorescent staining, which also demonstrated good adhesion and proliferation on all scaffolds.

#### Cytokine-loaded microspheres induce chondrogenic differentiation and expression of specific proteins in various layers of the composite scaffold

**Figure 5:**
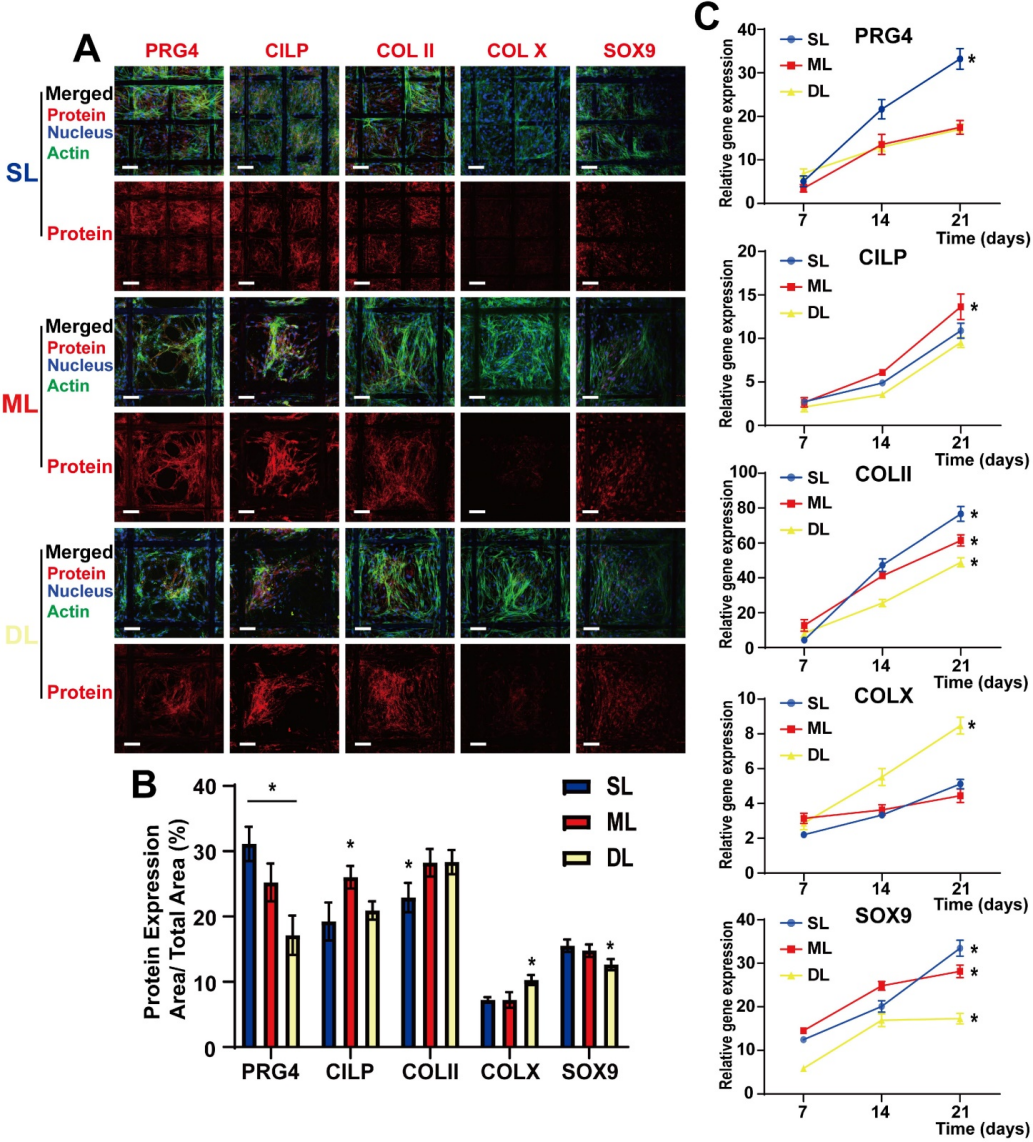
** Cytokine-loaded microspheres induce Chondrogenic differentiation and expression of specific proteins in various layers of the composite scaffold. A)** Confocal images of BMSCs in superficial layers (SL), middle layers (ML), and deep layers (DL), detected via immunofluorescence labeling after 21 days of cell seeding. Upper panels are “merged” (protein expression + nucleus + F-actin)” and lower panels are “protein expression.” **B)** Quantitative evaluation of the protein expression levels via ImageJ analysis of the immunofluorescence images. The results are expressed as protein expression area/total area × 100 (%); **C)** mRNA expression levels of PRG4, CILP, COLII, COLX, and SOX9, as determined by RT-PCR, at 21 d after cell seeding in various scaffold layers. (Scale Bar: 50 µm) (*P < 0.05 compared to other groups).

BMSCs were seeded on composite scaffolds, and the relative protein expression was observed after 21 days of culture and differentiation. Confocal microscopy performed after immunofluorescence staining, and real-time PCR (RT-PCR), were used to detect the relative protein and mRNA expression levels, respectively. BMSCs were observed to differentiate into chondrocytes following induction by sustained cytokine release from the microspheres (Figure 5A). As a marker protein of chondrocytes, SOX9 was expressed in all three layers, suggesting an overall chondrogenic differentiation; COLII, which induces cartilage formation, was also expressed in all three layers. Furthermore, the high COLII content in the ML helped support the cartilage structure, thereby improving the overall supportive capacity of the cartilage. The SL cells expressed a high level of PRG4 following induction by BMP-7, which is conducive to the lubrication of the cartilage surface, effectively reducing damage inflicted by cartilage friction. Additionally, CILP expression was induced by IGF-1 in the ML, demonstrating that this layer also has a sub-differentiated trend induced by IGF-1 (Figure 5A and 5B). These results were supported by those of rt-PCR analysis which showed that SOX9 was gradually upregulated over time, demonstrating that the slow-release cytokines in the composite scaffold gradually induced cartilage differentiation. Additionally, COLII expression was upregulated in all layers and had the highest expression in the SL, which may be related to the denser structure of the SL, resembling healthy cartilage. PRG4 expression was higher in SL than in ML and DL, while CILP expression was higher in ML compared to the SL and DL; this was also beneficial to the structural support of the entire cartilage (Figure 5C). These distinctions among components of various layers are conducive to the biological function of the scaffold.

#### *In vivo* cartilage injury repair following implantation of composite scaffolds

**Figure 6:**
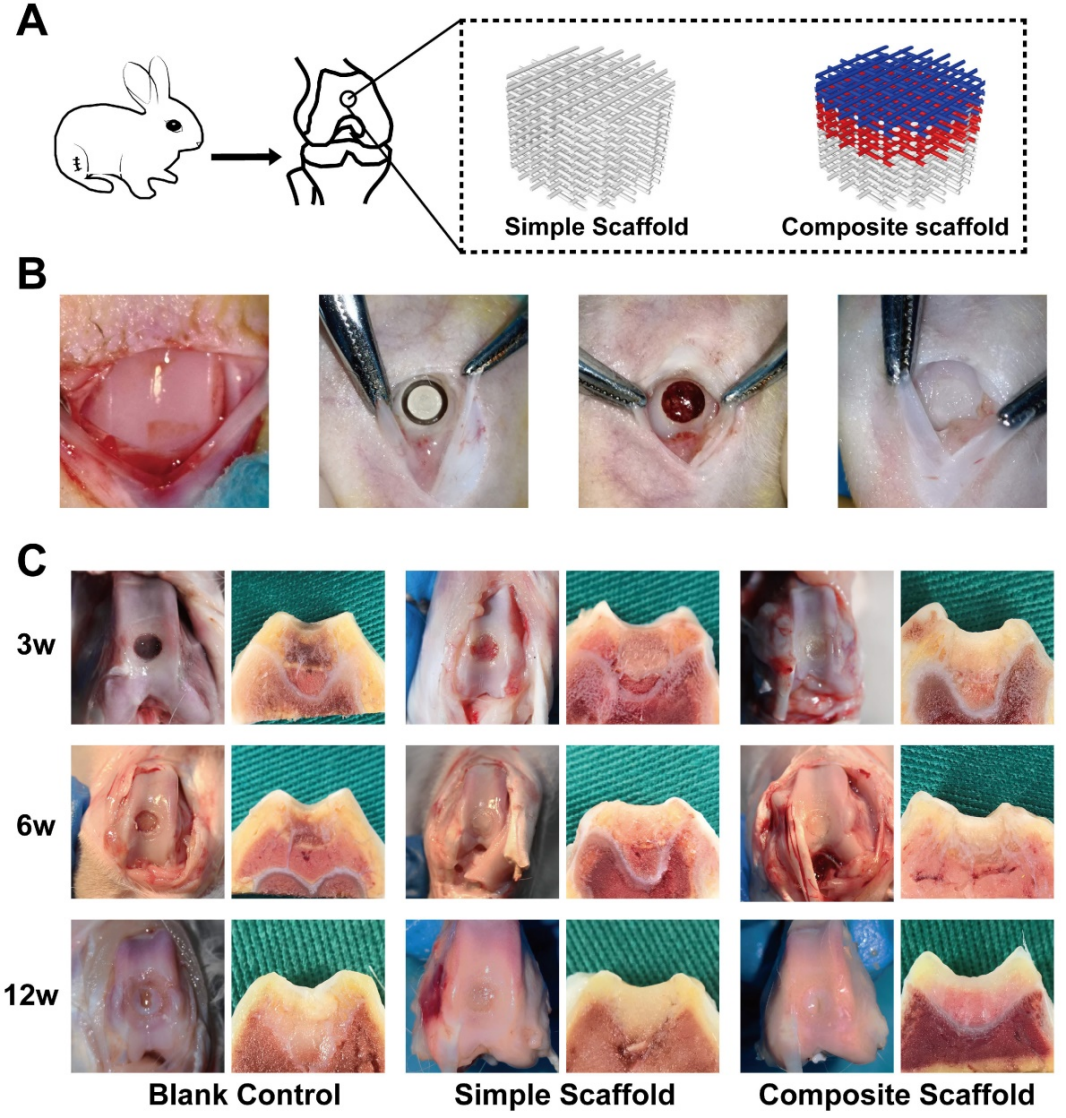
** Gross view and cross-sectional observation on operative day and after harvesting, respectively. A)** Schematic diagram of animal experiments; **B)** Gross view of surgical procedure; **C)** Gross view (left panel) and a cross-sectional view (right panel) of the blank control group, simple scaffold group, and composite scaffold group after 3, 6, and 12 w of scaffold implantation.

In this part of the study, a rabbit femoral intercondylar osteochondral injury model was used (Figure 6A). Cartilage injury repair was evaluated at 3, 6, and 12 w after transplantation of various scaffolds. The injury was poorly repaired in the blank control group at 3 and 6 w, with the damage visible with scar tissue apparent in the interior (Figure 6B). In both experimental groups, although the injury was covered by 6 w, a clear boundary was visible with the surrounding tissue. Fillers and surrounding decomposition profiles were also visible in the cross-section. At 12 w, defects were still observed in the injured area of the blank group, however, the cartilage on the surface had regenerated in the center. In the simple scaffold group, the cartilage lesion was covered however, the surface was not smooth. Meanwhile, cross-section of the tissue demonstrated that the scaffold was well integrated with the surrounding tissues. Alternatively, the composite cartilage group formed smooth cartilage on the surface at 12 w, and the cross-section showed that the scaffold was well integrated with the surrounding tissue (Figure 6B).

**Figure 7:**
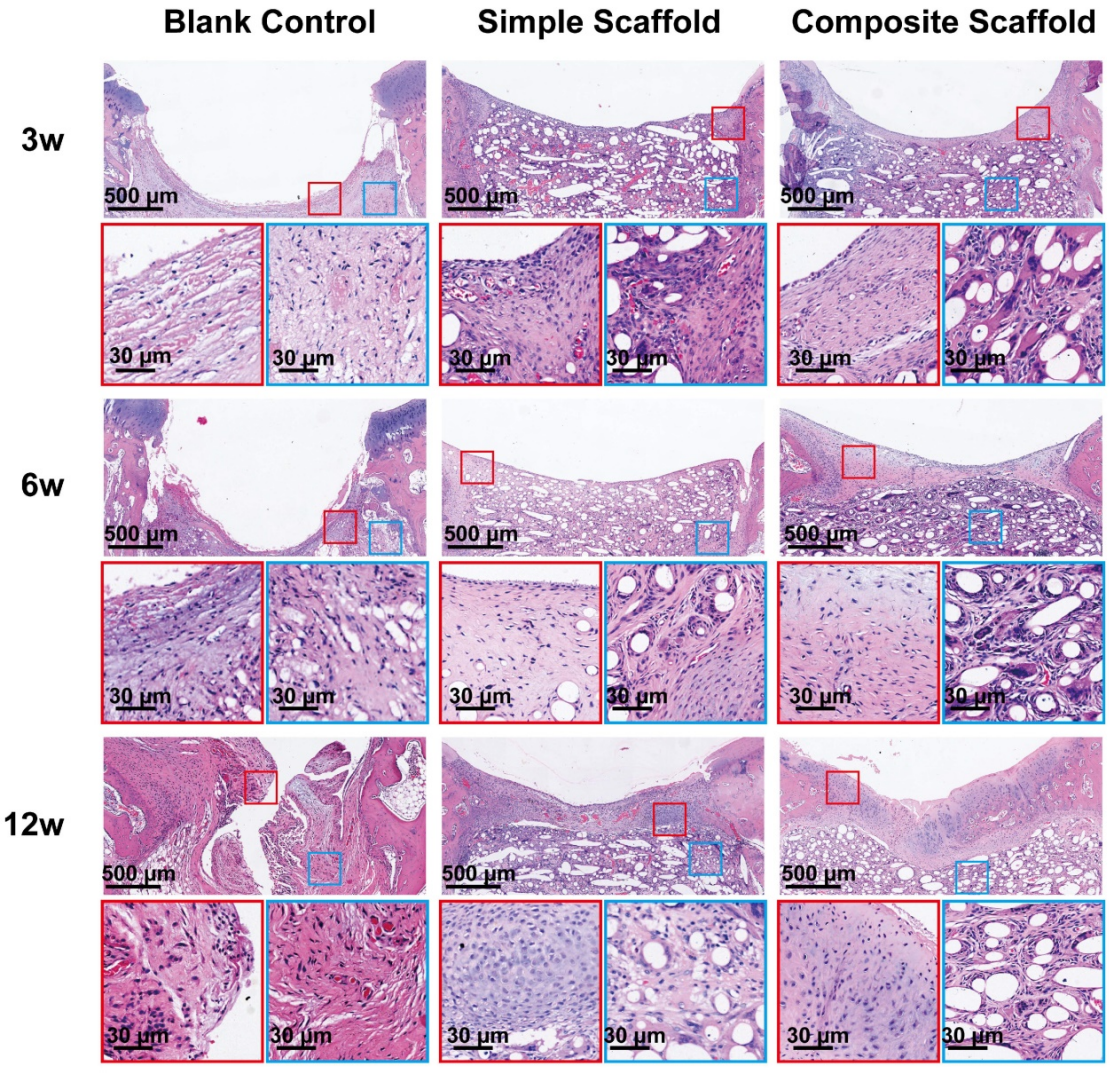
Microscopic appearance in the HE stained blank control, simple scaffold, and composite scaffold groups at 3, 6, and 12 w.

To further evaluate cartilage healing, the injured site was cross-sectioned and HE staining performed. Similar to the gross observations, the injured cartilage in the blank control group had visible defects at 12 w and was accompanied by structural disorder (Figure 7). The boundary between the injured area and the surrounding tissue was still apparent under the HE staining of the blank control group. On the other hand, in the experimental groups, due to the filling of the scaffold, an improved environment for attachment was provided to the cells, promoting development of tissue with normal structure and shape. At 12 w, the cartilage tissue was regenerated in the SL of the composite scaffold group, and a normal tissue shape was observed. However, since the PCL material dissolved during the sectioning process, voids were apparent in the images. The morphology of the repaired tissue was also confirmed via Sirius red staining (Supplementary Figure 5), which demonstrated that the collagen on the surface of the composite scaffold at 12 w was significantly better than in the other two groups. Therefore, adding cytokine-loaded microspheres served to significantly enhance the biological activity of the scaffold.

**Figure 8:**
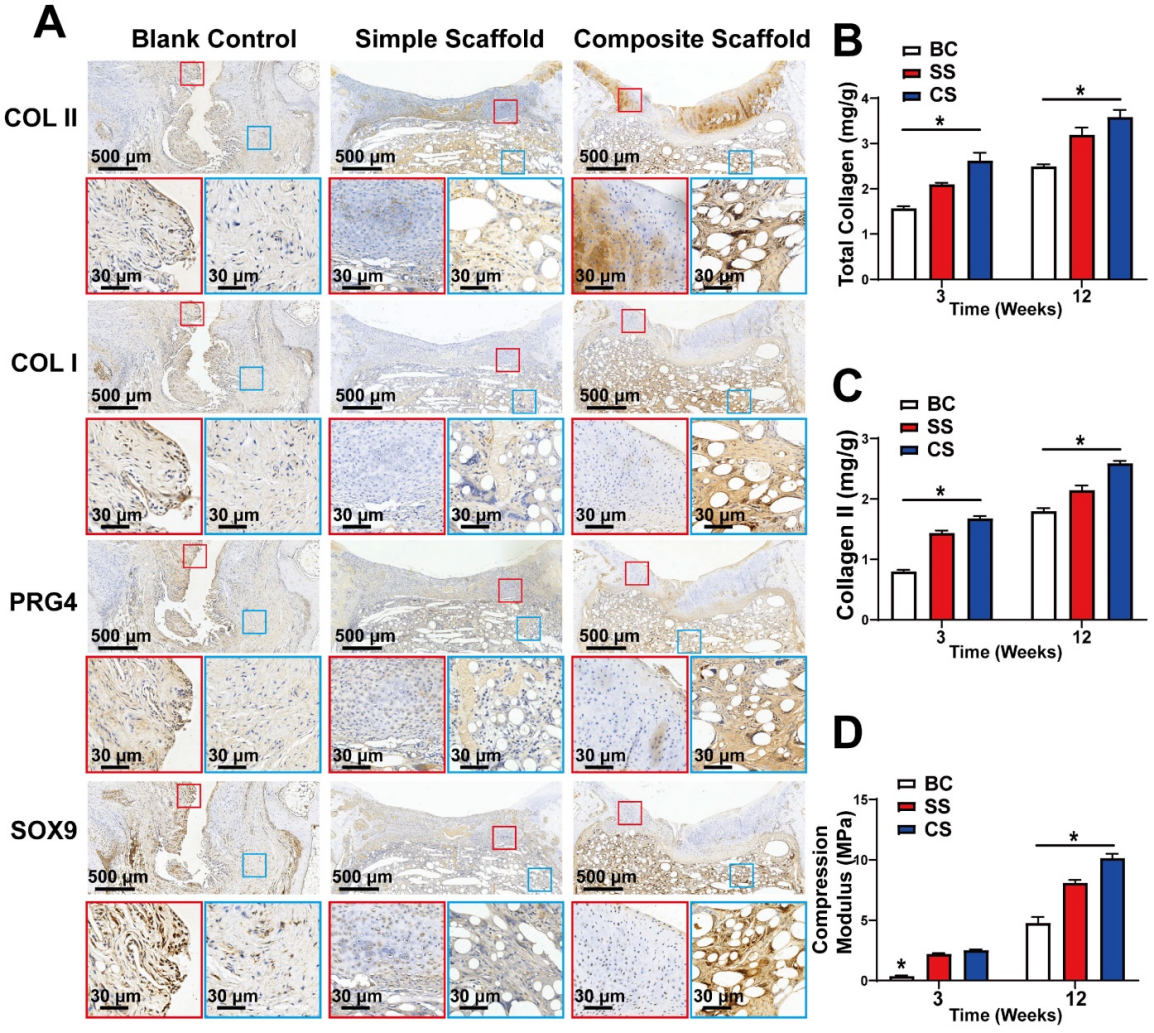
** Composition and mechanical analysis of regenerated cartilage. A)** Immunohistochemical analysis of COLII, COLI, PRG4, and SOX9 expression levels in the regenerated cartilage of the blank control group, simple scaffold group and composite scaffold group at 12 w; **B)** Total collagen content, **C)** COLII content, and **D)** Compression modulus of the regenerated cartilage in various groups, as indicated, at 3 w and 12 w. (*P < 0.05 compared to other group).

Immunohistochemical analysis of the cartilage tissue was also performed 12 w after injury and scaffold implantation to evaluate the expression levels of COLII, COLI, PRG4, and SOX-9. SOX-9 is a cartilage marker, while PRG4 is primarily expressed by superficial cartilage. Hence, increased expression of PRG4 indicates superior resistance to friction. In the composite scaffold group, a high expression of cartilage components was observed, and COLII expression was higher than in the simple scaffold or blank control groups (Figure 8A). The COLII component gradually decreased from the SL with increased depth, which mimicked the structure of healthy cartilage. In addition, there was a high expression of PRG4 protein in the SL of the composite scaffold group, indicating that the repaired cartilage was rendered smoother and had better friction resistance. The expression of COLI and SOX9 was also higher in the composite scaffold group compared to the other groups. Overall, the composite scaffold group had a protein expression profile similar to that of healthy cartilage.

Finally, the tissue of the regenerated section was extracted for component and mechanical analysis, as well as for quantitative analysis of total collagen and COLII content. At both 3 and 12 w, the collagen content of the composite scaffold group was higher than that of the other two groups (Figure 8B, C). Moreover, the compressive modulus test revealed that the composite scaffold group had better mechanical properties compared to the other groups (Figure 8D).

## Discussion

Different methods and materials have been used to construct suitable implants for clinical repair of damaged cartilage 21, 36, 37. However, these methods have fallen short in their ability to reconstruct the complex structure of adult cartilage tissue. Although studies have reported the successful preparation of bone-cartilage biphasic scaffolds for osteochondral damage 13, 38, 39, these artificial scaffolds were unable to mimic the complex, multi-layered structure of natural cartilage. In this study, MEW and Inkjet printing were combined to construct a new 3D printed system to promote the regeneration of cartilage tissue.

Scaffolds play a crucial role in regenerative medicine by providing the appropriate 3D environment for cell adhesion, proliferation, and matrix production 40, 41. An ideal scaffold should, therefore, be similar in function and structure to normal tissue and support the differentiation and formation of different tissue morphologies. Compared to the uniformity of traditional manufacturing processes, MEW adds an electrostatic field on the melt extrusion printing, which stretches the material and further reduces the fiber diameter from 200 μm to 1-20 μm, resulting in increased accuracy in printing. Moreover, as the fiber diameter decreases, the structure changes with increased porosity and reduced impact on tissues, resulting in decreases in the overall improved mechanical properties, which makes it more suitable for the repair of cartilage and other tissues 42-44. Previous studies on cartilage repair have shown that 200 μm pores are more suitable for chondrocytes in a deeper layer, while 100 μm pores are beneficial for the SL 45-47. By switching the printing cavity, as in the method described herein, it is possible to print different materials simultaneously to build a more sophisticated structure that more closely micks the physiological tissues.

Based on the structure, moldability and biocompatibility of the material, and the desired mechanical properties of the scaffold, polycaprolactone (PCL) was selected as the basic material for printing. PCL is a degradable material commonly used in tissue engineering as it has strong stability, good biocompatibility, and easy processing 48, 49. To ensure compatibility of the scaffold DL with the subchondral bone, PCL was mixed with 10% HA as the deep layer scaffold material. During the printing process, the disposable preparation was completed by replacing the barrel and the print head. By mixing HA with PCL, the compressive strength of the scaffold was further improved, which is more suitable for the performance of the cartilage calcification layer, while reducing the tensile strength.

The PCL scaffold as a whole supports the frame with its sole biological function focused on providing an appropriate porous environment for cells, it does not have biological functions. Therefore, to further improve the repair effect, cytokines were used to induce cartilage differentiation of BMSCs that are exuding from the site of injury. The cytokines used were TGF-β1, BMP-7, and IGF-1. To ensure that the cytokines were properly packaged and could be maintained for an extended period, PLGA was used to make cytokine-loaded microspheres. Inkjet spraying was then employed to spray the cytokine-loading microspheres in appropriate positions. To make the microspheres more stable, a slightly soluble solution was used to facilitate their firm adhesion to the scaffold. Thus, a 3D printed composite scaffold with integrated biological functions was formed.

The prepared biologically active 3D printed composite scaffolds mimicked the composition of layered performance of normal cartilage tissue. The composite scaffold was used to provide support for BMSCs, which were induced to proliferate and differentiate by the sustained release of cytokines from PLGA microspheres in the composite scaffold 50. The heterogeneous cytokine environment in different layers of the scaffold then induced the stratified BMSC differentiation into various cartilage components, which in turn promoted biomimetic tissue repair. The composite scaffold, therefore, can provide an improved strategy for cartilage damage repair.

TGF-β1 can promote chondrogenic differentiation and SOX-9 expression, however, it is less effective in mature chondrocytes. SOX-9 is a marker for chondrogenic differentiation in BMSCs 51, 52. In the current study, the expression of PRG4 was higher in the SL than in the ML and the DL. This difference is likely due to the combined application of TGF-β1- and BMP-7-containing microspheres in the SL. Studies have shown that inactivation of mouse BMP receptors results in SOX-9 expression loss. Meanwhile, BMP-7 can significantly induce PRG4 and further promote the effect of TGF-β1 26. Furthermore, the high expression of SOX-9 after TGF-β1 and BMP-7 treatment may involve signaling through the SMAD pathway 24, 53. These results underscore the importance of the combination application of BMP-7 and TGF-β1 to guide the differentiation of BMSCs into surface area phenotypes 10, 54-56.

In natural cartilage, collagen is arranged parallel to the cartilage surface, a pattern that was observed in our study results. During the shaping and development of human tissues, the collagen content and direction of the surface area are adjusted by external loads, forming a structure parallel to the cartilage surface. In our study, the arrangement of collagen also showed this characteristic 57, 58.

The ML of our scaffold contained microspheres releasing IGF-1, which is an anabolic growth factor that interacts with TGF-β1 and directly affects the upregulation of COLII and AGC in proliferative chondrocytes. IGF-1 has been shown to stimulate proteoglycan and collagen synthesis in pre-mast chondrocytes *in vitro* and *in vivo*. In an *in vivo* study that reported on the effects of IGF-1 on cartilage tissue development, cartilage-specific IGF-1 knockout mice died shortly after birth and showed disordered chondrocyte numbers. In addition, exposing chondrocyte grafts to IGF-1 could significantly increase collagen synthesis. Meanwhile, IGF-1 can stimulate PRG4 synthesis in pre-mast chondrocytes *in vitro* 59-61.

The DL had a higher expression of COLX, which could be attributed to the combined effect of TGF-β1 and HA. Khanarian *et al.* observed a higher expression of COLX in pre-mast chondrocytes taken from DLs of bovine articular cartilage in mixed HA culture. Since the microfracture opens the subchondral bone, the scaffold DL is able to communicate with the bone marrow. Therefore, the relative oxygen content in the DL would be higher, which serves to reduce the risk of hypoxia and promote the differentiation of BMSC in this layer into deep cartilage. Furthermore, the cells in the DL were in contact with HA, which might also provide a cartilage-inducing effect. In addition, the compression modulus of PCL with HA was higher, compared to the simple PCL scaffold, which could also be conducive to the formation of cell calcification as the 3D HA-fiber scaffold structure is reported to enhance endochondral ossification of bone marrow stem cells 62-64.

In summary, the high precision MEW and Inkjet printing technology were successfully combined to construct a bioactive cartilage repair scaffold that was applied for the repair of cartilage defects. However, a few shortcomings must be addressed in future research. First, although the good biocompatibility and print formability make PCL the first choice for MEW, the degradation rate and biological activity are not adequate, which may restrict the overall repair efficiency. Second, although partial repair of cartilage injury was achieved, the regenerative potential needs to be further improved and strengthened. Nevertheless, the establishment of this 3D printing system provides a new basis to construct soft-tissue repair scaffolds.

## Conclusion

This study demonstrates a novel method of scaffold production for cartilage tissue repair. By combining MEW with Inkjet printing, the relevant cytokine-packaged microspheres were accurately loaded on a higher-precision 3D printed scaffold. *In vitro*, the composite scaffold had excellent biocompatibility, balanced degradation, and provided support for cells. The sustained release of microspheres on the scaffold provided corresponding cytokines at specific locations of the scaffold to induce directional differentiation of cells. *In vivo* experiments demonstrated that the scaffold filled the injury site and promoted partial repair of the injury. Overall, this study provides a new printing system for 3D scaffolds mimicking typical cartilage components to boost injury repair.

## Statement of Significance

Recently, considerable attention has been paid to applying 3D printing in biomedicine, however, the accuracy of 3D printing remains insufficient for complex biological applications. Here, we coated a MEW printed PCL-based scaffold, with the corresponding cytokine-containing microspheres using Inkjet, to construct a cartilage repair scaffold with sound mechanical and biological effects. The technology described herein can be applied to other bio-medical applications besides cartilage repair, including vascular and tracheal scaffolds. This study provides an efficient, stable, and novel tool for the application of 3D printing technology for tissue engineering.

## Supplementary Material

Supplementary figures and tables.

## Abbreviations

BMP-7: bone morphogenetic protein 7
BMSCs: bone marrow mesenchymal stem cells
CILP: cartilage intermediate layer protein
COLII: collagen type II
COLI: collagen type I
DCM: methylene dichloride
DL: deep layer
FDM: fused deposition molding
HA: hydroxyapatite
IGF-1: insulin-like growth factor-1
MEW: Melt Electro-Writing
ML: middle layer
OA: Osteoarthritis
PCL: polycaprolactone
PLGA: poly D,L-lactic-co-glycolide
PRG4: proteoglycan 4
PVA: polyvinyl alcohol
RT-PCR: quantitative reverse transcription-polymerase chain reaction
SEM: scanning electron microscopy
SL: surface layer
SOX9: Sox-9
TGF-β1: transforming growth factor-beta 1
