# Supplementary material for: Preparation of high precision multilayer scaffolds based on Melt Electro-Writing to repair cartilage injury
Source: Theranostics. 2020 Aug 13;10(22):10214–30. doi: 10.7150/thno.47909 (PMC7481411; doi:10.7150/thno.47909)
Supplement: Supplementary file 1 — Supplementary figures and tables. [file thnov10p10214s1.pdf]

Supplementary Materials

Supplementary Table 1. Forward and reverse primers used for quantitative rt-PCR

Gene	Species	Primer sequence	Product length
PRG4	*Oryctolagus*	F:5′-CATCTCCACCTCGCAGAATCA-3′<br>R:5′-TGCTGGATGTTGCCACCTCTC-3′	275
CILP1	*Oryctolagus*	F:5′- TGATGGTCATAGCACCCGA-3′<br>R:5′-AACATGCCGTAGGTCCGAAG-3′	305
COLII	*Oryctolagus*	F:5′-CCACGCTCAAGTCCCTCAACAA-3′<br>R:5′-TCCAGTAGTCACCGCTCTTCCA-3′	129
COLI	*Oryctolagus*	F:5′-AAGCCGGTCGTGATGGCAA-3′<br>R:5′-CACCGACGGGACCAATAGAACC-3′	207
SOX9	*Oryctolagus*	F:5′-AAGATGACCGACGAGCAGGAGA-3′<br>R:5′-TGTTCTTGCTGGAGCCGTTGAC-3′	280
β-Actin	*Oryctolagus*	F:5′-TCTTCCAGCCCTCCTTCCTG-3′<br>R:5′-CGTTTCTGCGCCGTTAGGT-3′	142

CILP1: cartilage intermediate layer protein 1; COLI: collagen type I; COLII: collagen type II; F: forward; PRG4: proteoglycan 4; R: reverse

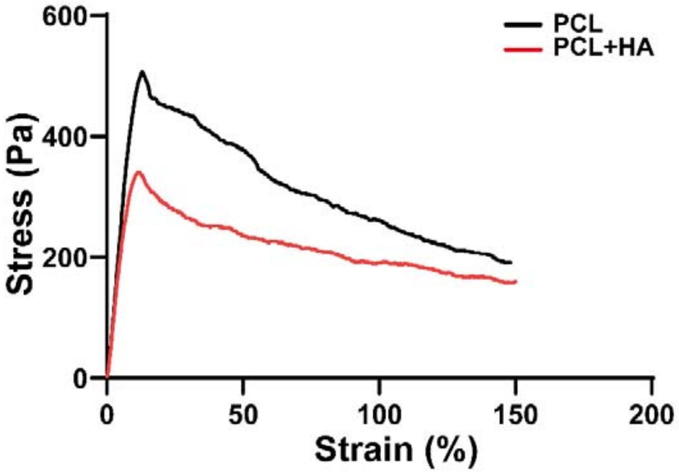

Supplementary Figure 1. Tensile modulus test curve of the scaffold.

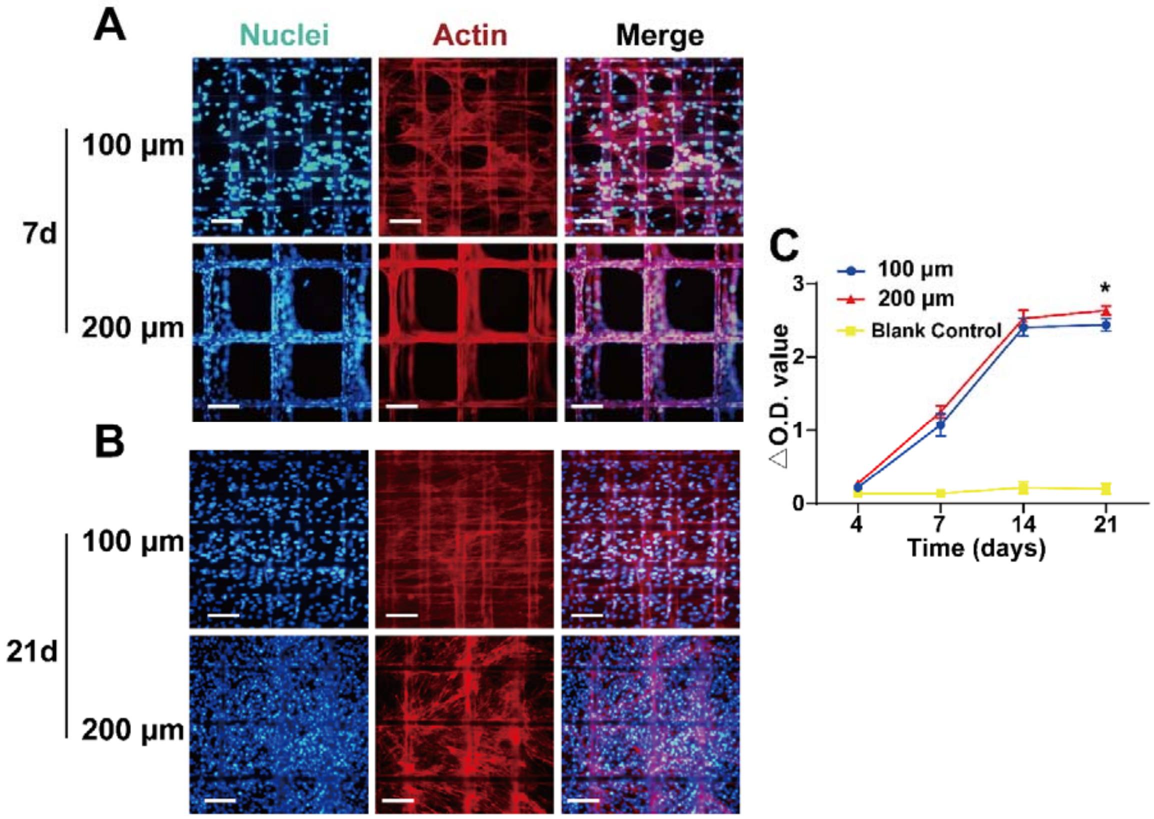

Supplementary Figure 2. BMSC adhesion and proliferation on the pure PCL scaffolds. A & B) BMSC adhesion and proliferation on 100 μm and 200 μm scaffolds at 7 d and 14 d, respectively. C) CCK-8 assay results showing proliferation rates of the BMSCs on scaffolds at various time points (Scale Bar: 100 μm) (*P < 0.05).

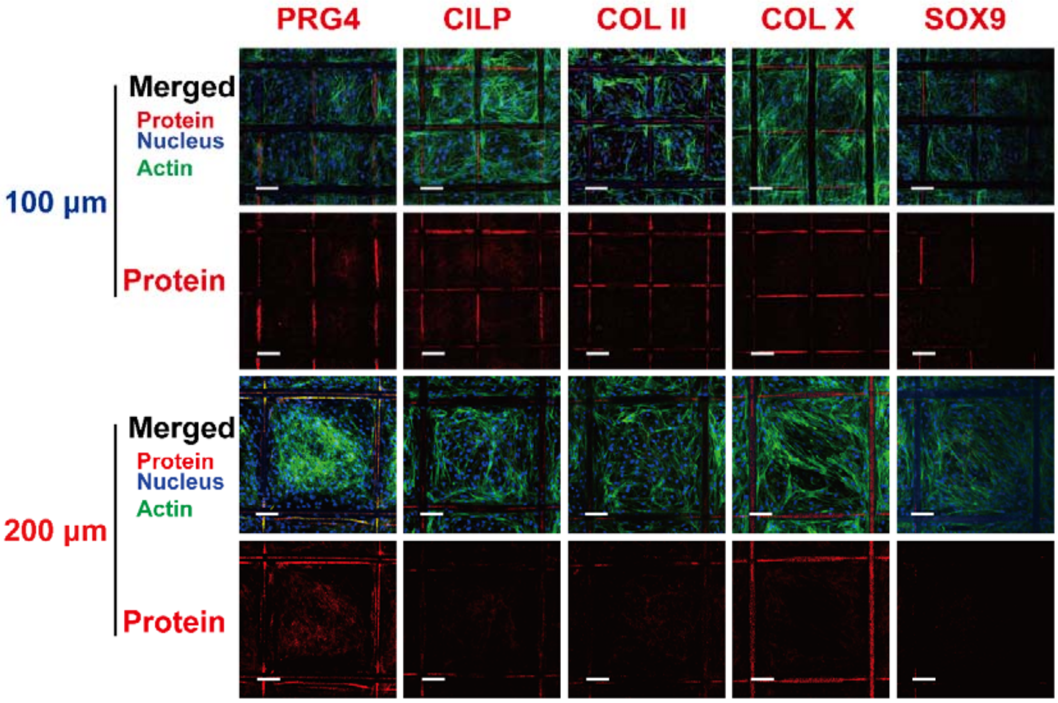

Supplementary Figure 3. Confocal images of BMSCs in pure PCL scaffold group immunofluorescence labeling after 21 days of cell culture (Scale Bar: 50 μm). CILP1: cartilage intermediate layer protein 1; COLI: collagen type I; COLII: collagen type II; PRG4: proteoglycan 4.

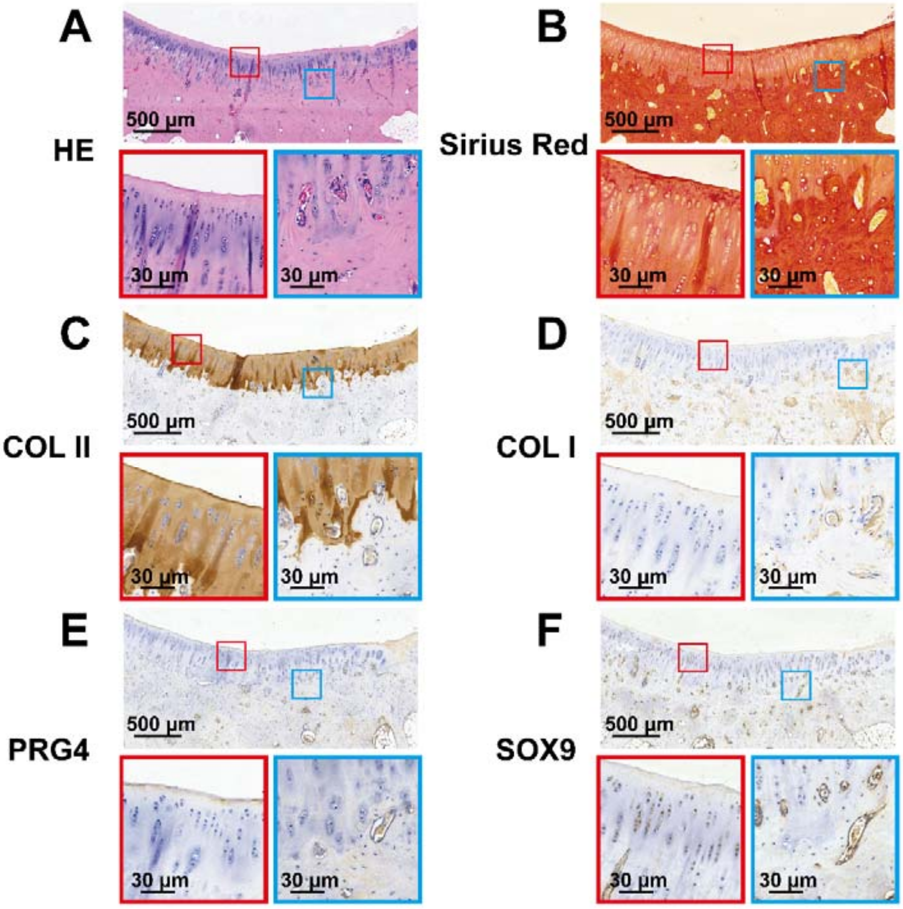

Supplementary Figure 4. Microscopic appearance in the HE, Sirius red staining and immunohistochemical staining of COLII, COLI, PRG4, and SOX9 of the native cartilage.

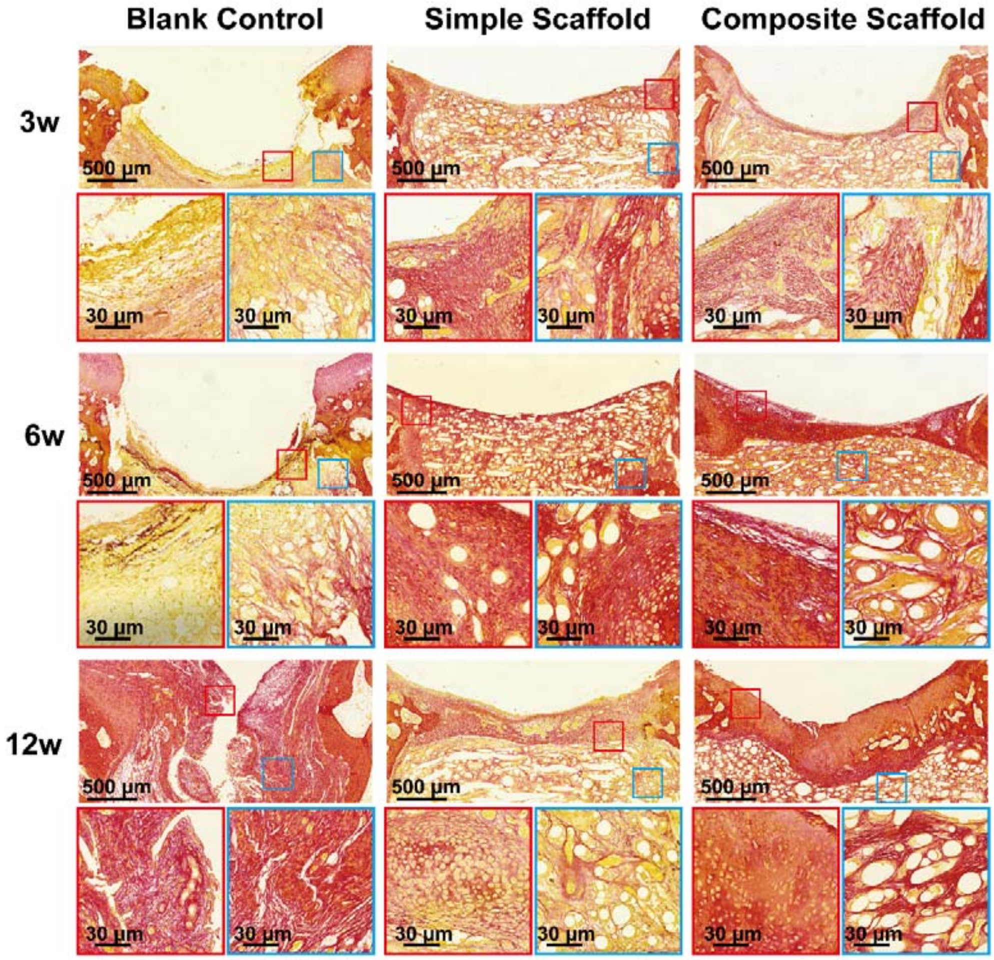

Supplementary Figure 5. Microscopic appearance in the Sirius red staining of the blank control group, simple scaffold group, and composite scaffold group at 3, 6, and 12 weeks.

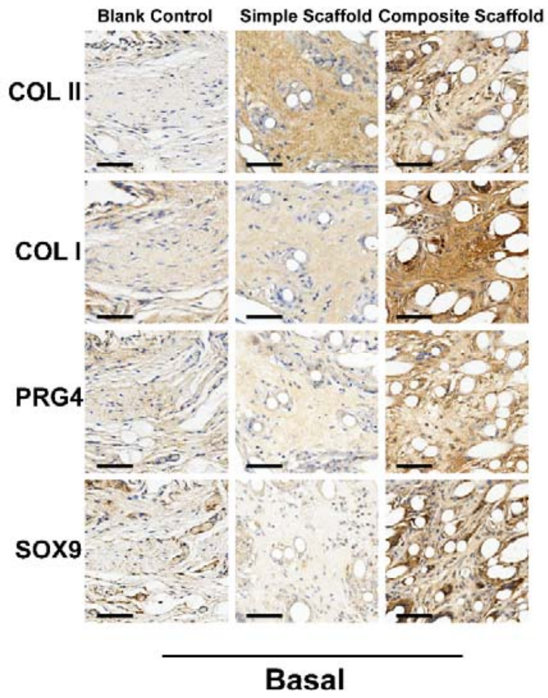

Supplementary Figure 6. Immunohistochemical analysis of COLII, COLI, PRG4, and SOX9 expression levels at the basal area of the regenerated cartilage of the blank control group, simple scaffold group, and composite scaffold group at 12 weeks (Scale Bar: 30 μm).

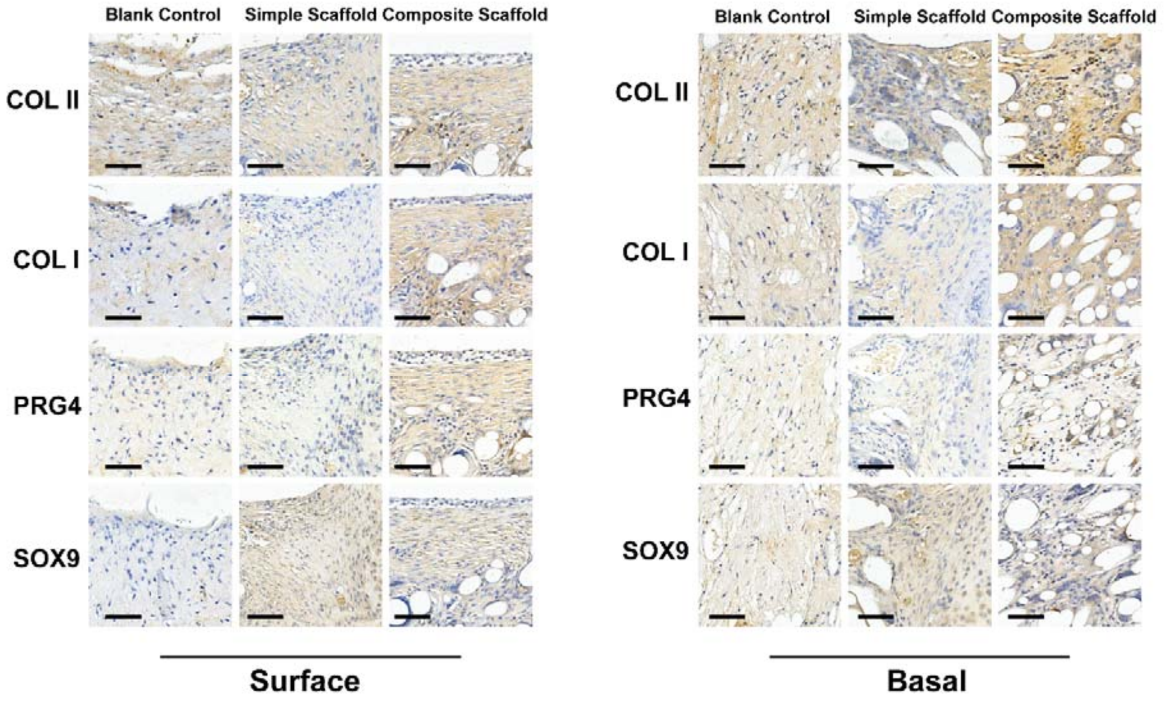

Supplementary Figure 7. Immunohistochemical analysis of COLII, COLI, PRG4, and SOX9 expression levels at the surface and basal areas of the regenerated cartilage of the blank control group, simple scaffold group, and composite scaffold group at 3 weeks (Scale bar: 30 µm).

#
